# Supplementary material for: Unravelling the role of redox active sites in nitrogen doped cerium oxide for associative ammonia decomposition
Source: Nat Commun. 2026 Mar 13;17:3892. doi: 10.1038/s41467-026-70330-5 (PMC13125523; doi:10.1038/s41467-026-70330-5)
Supplement: Supplementary file 1 — Supplementary Information [file 41467_2026_70330_MOESM1_ESM.pdf]

Supporting Information for

Unravelling the Role of Redox Active Sites in Nitrogen Doped Cerium Oxide for Associative Ammonia Decomposition

D. Ye et al.

This file includes:

Figures S1 – S26

Tables S1 – S4

Supplementary Discussions 1 – 6

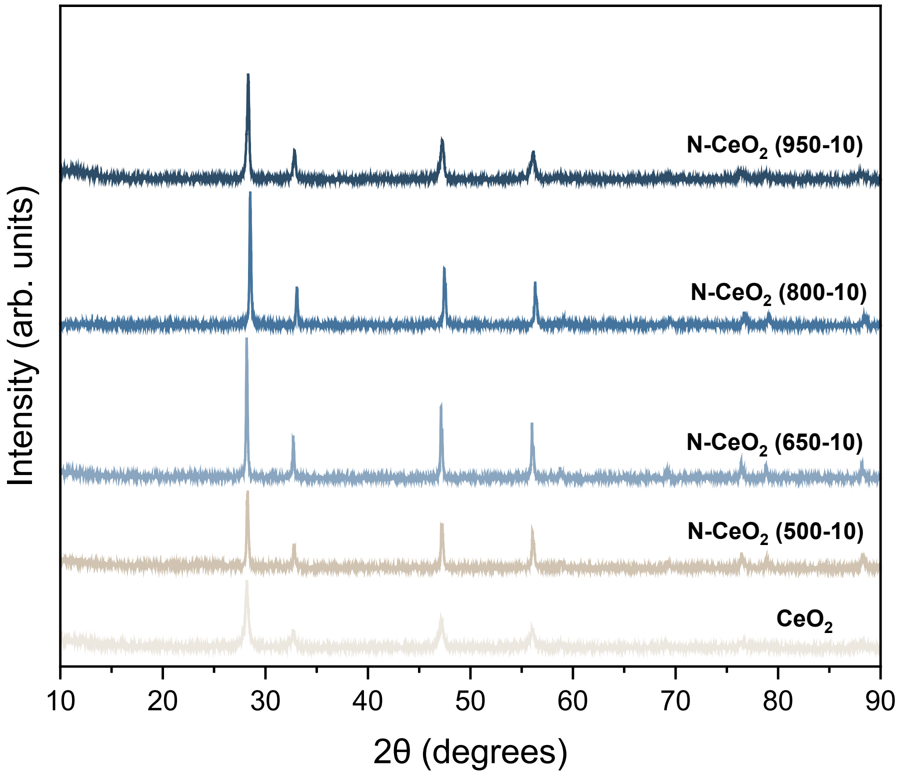

Fig. S1 X-ray diffraction pattern of CeO_2 and N doped CeO_2 sample treated under NH_3 flow at different temperatures.

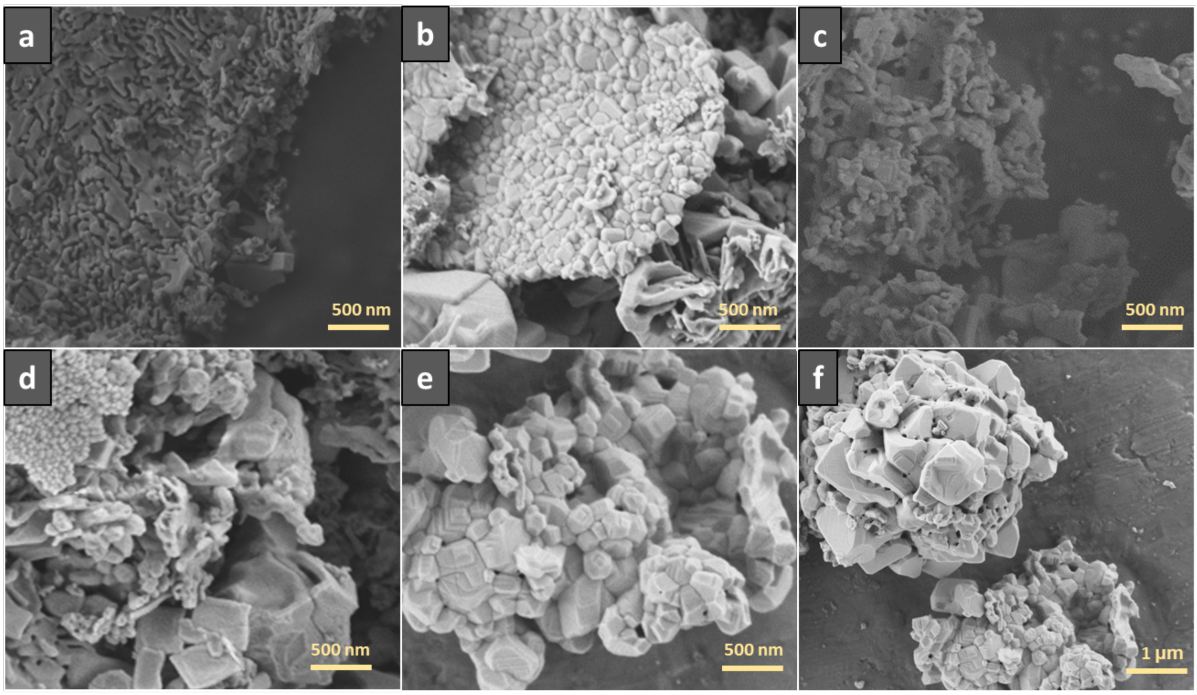

Fig. S2 Scanning electron microscopy of Cerium oxide-based supports. a. CeO_2 b. N-CeO_2 (500-10) c. N-CeO_2 (650-10) d. N-CeO_2 (800-10) e. N-CeO_2 (950-10) f. N-CeO_2(950-10) at Magnification = 5K.

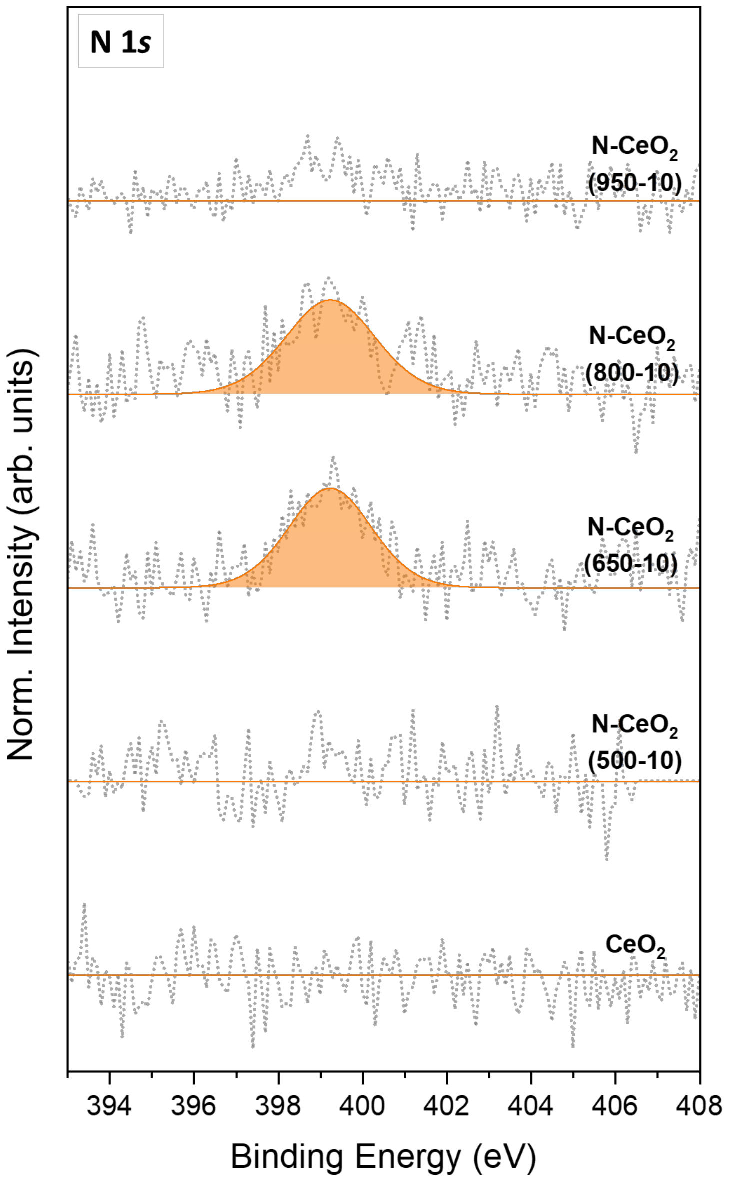

Fig. S3 High resolution X-ray photoelectron spectroscopy results for the N 1*s* orbitals of the cerium oxide and N-doped cerium oxide supports.

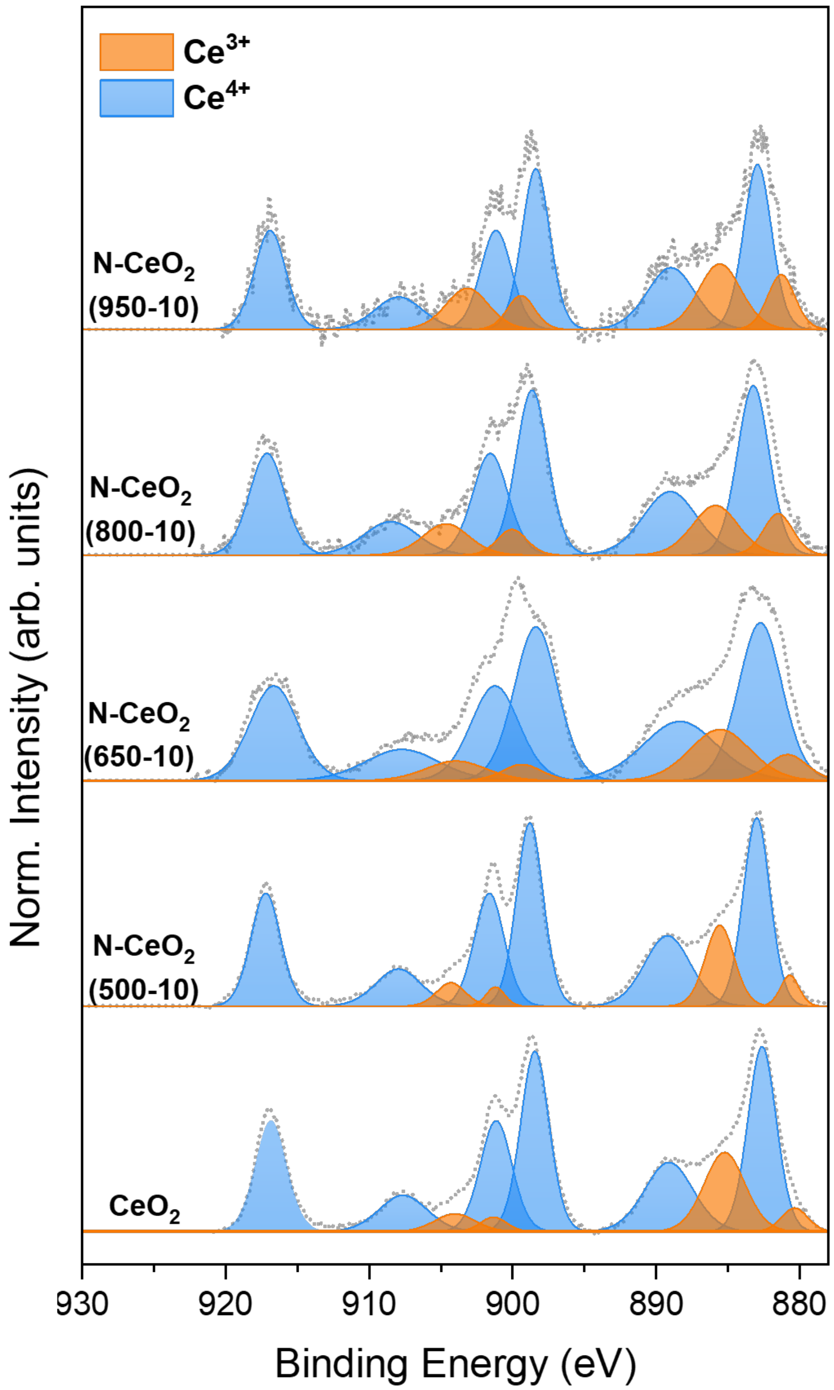

Fig. S4 High resolution X-ray photoelectron spectroscopy results for the Ce 3*d* orbitals of the cerium oxide and N-doped cerium oxide supports. The high-resolution Ce 3*d* orbital spectrum is highly complicated and is deconvoluted into 10 peaks. 6 distinct peaks, denoted as u0 (901.0 eV), u′ (907.3 eV), u″ (916.6 eV), v0 (882.4 eV), v′ (888.6 eV), and v″ (898.2 eV), are associated with Ce^{4+}. There are 4 further characteristic peaks, namely u0 (900.6 eV), u′ (903.6 eV), v0 (880.1 eV) and v′ (884.9 eV) representing Ce^{3+}.[1]

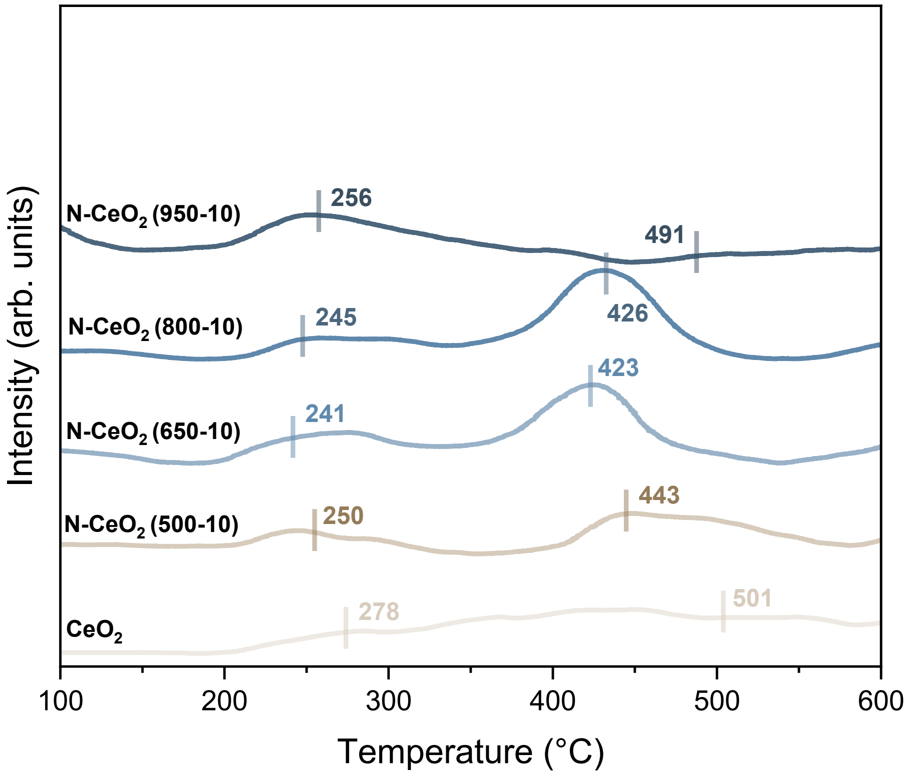

Fig. S5 H_2-TPR spectrum of the cerium oxide and N-doped cerium oxide supports.

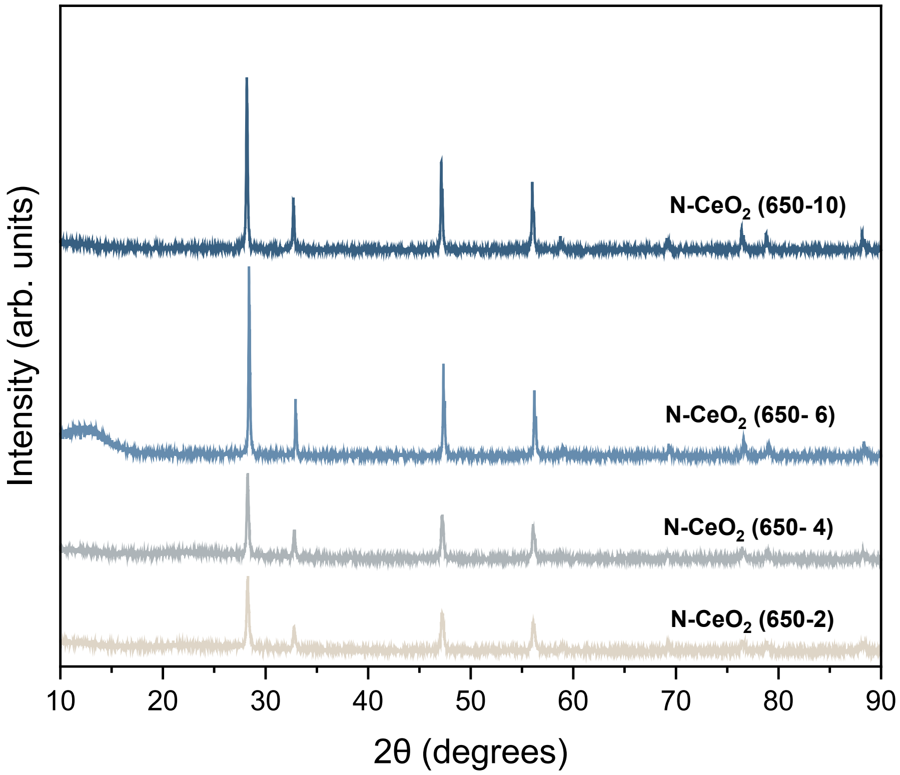

Fig. S6 XRD pattern of the N-doped cerium oxide supports calcined at 650 °C with different annealing durations.

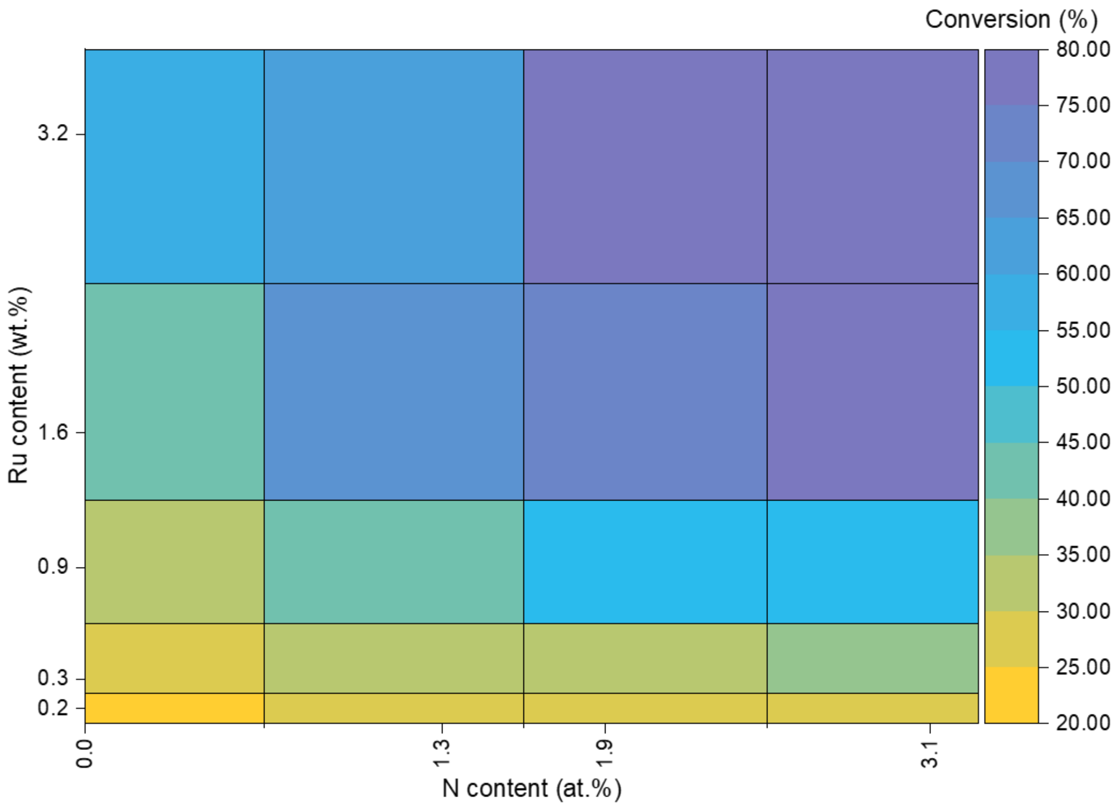

Fig. S7 Catalytic evaluation of catalysts with different Ru loading (wt.%) and N content (at.%) on ceria support. All measurements were conducted at 450 °C and WHSV = 30,000 ml g_{cat}^{-1} h^{-1}.

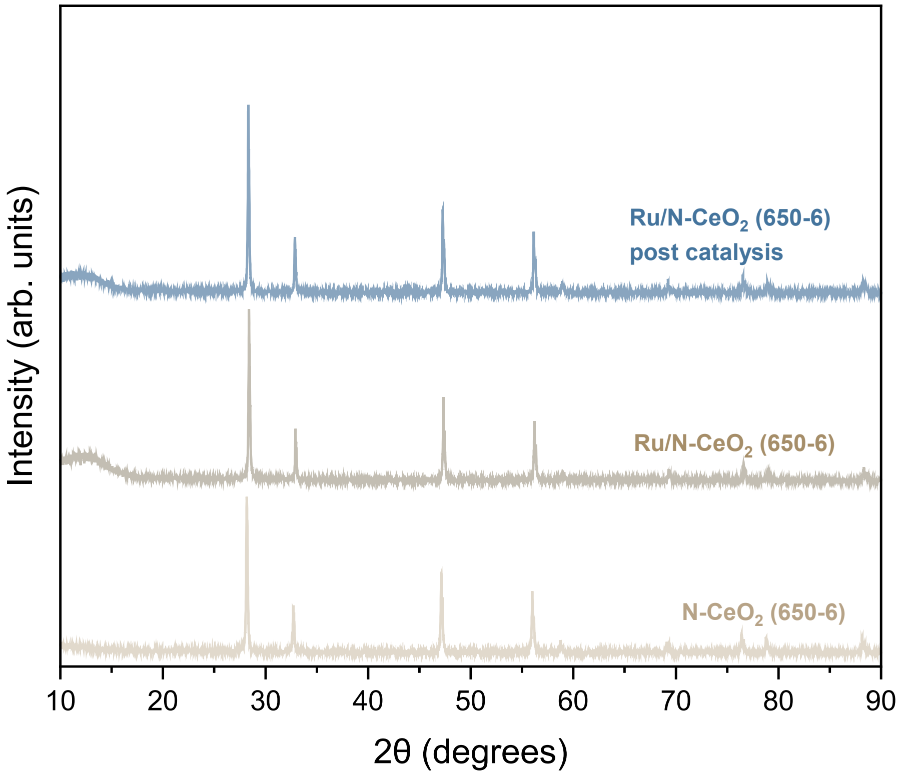

Fig. S8 XRD pattern of the N-CeO_2 (650-6), after Ru impregnation and post catalysis. No significant change in its lattice structure is observed.

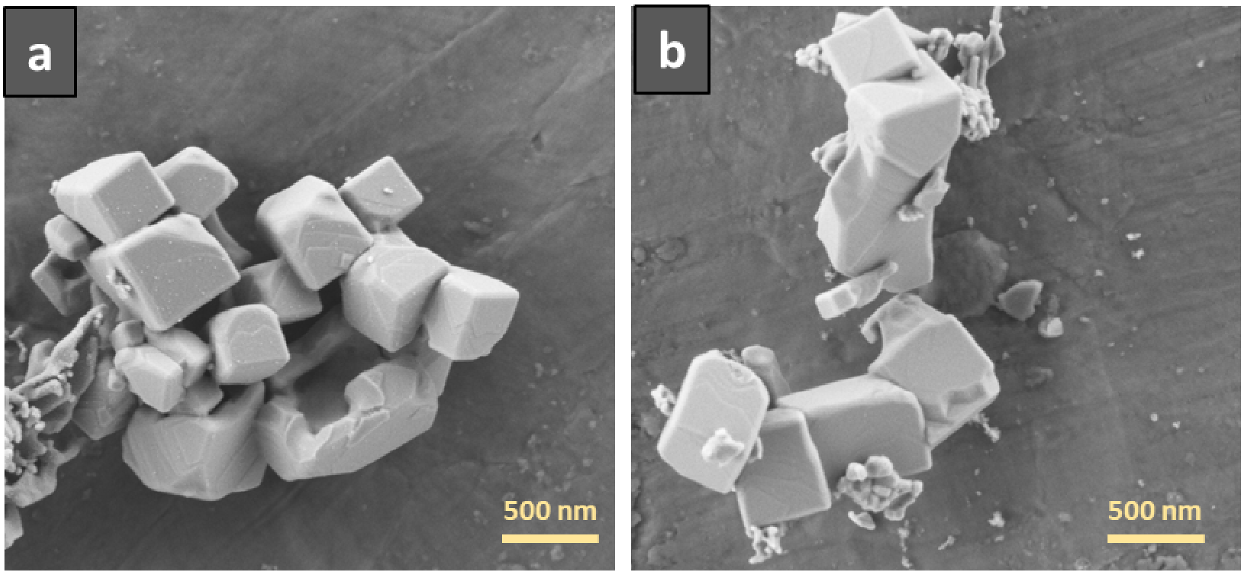

Fig. S9 Scanning electron microscopy of a. Ru/N-CeO_2 (650-6) b. Ru/N-CeO_2 (650-6) post catalysis.

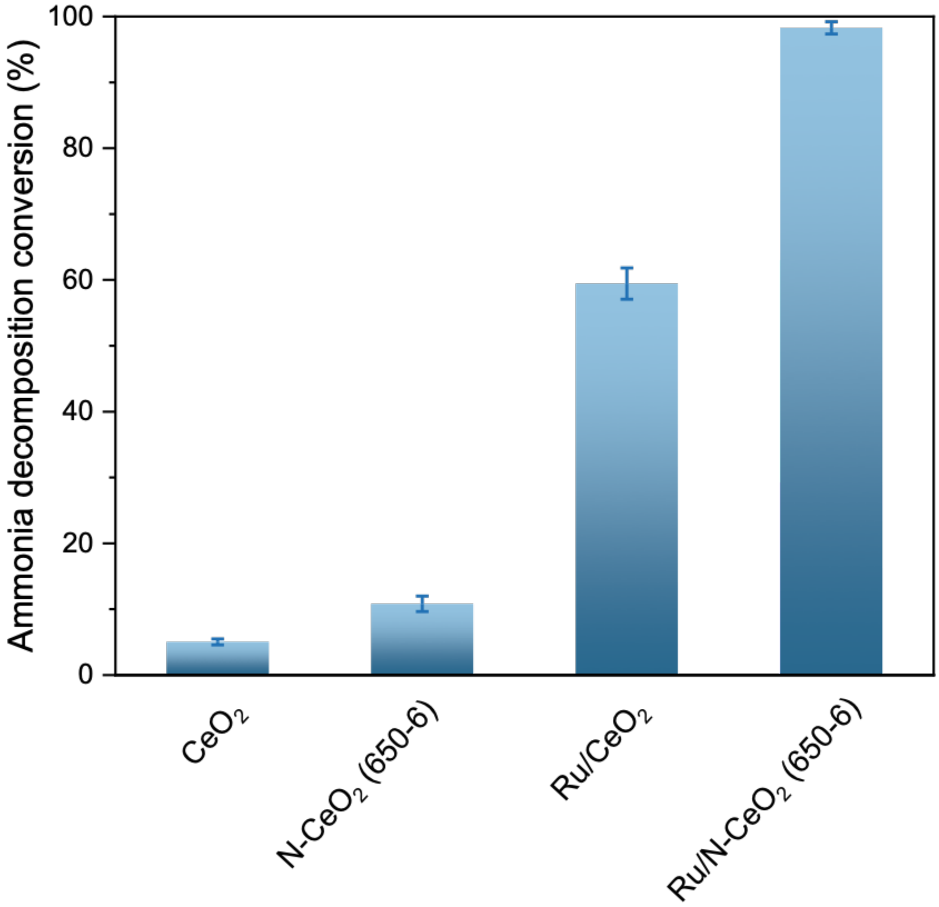

Fig. S10 Ammonia decomposition of pristine cerium oxide, optimised N-doped cerium oxide supports, and the Ru impregnated catalysts. The conversion taken at 500 °C and WHSV = 30,000 ml g_{cat}^{-1} h^{-1}. Error bars represent the mean ± standard deviation from 3 independent measurement. Each measurement was conducted under identical conditions using a freshly prepared batch of catalyst sample.

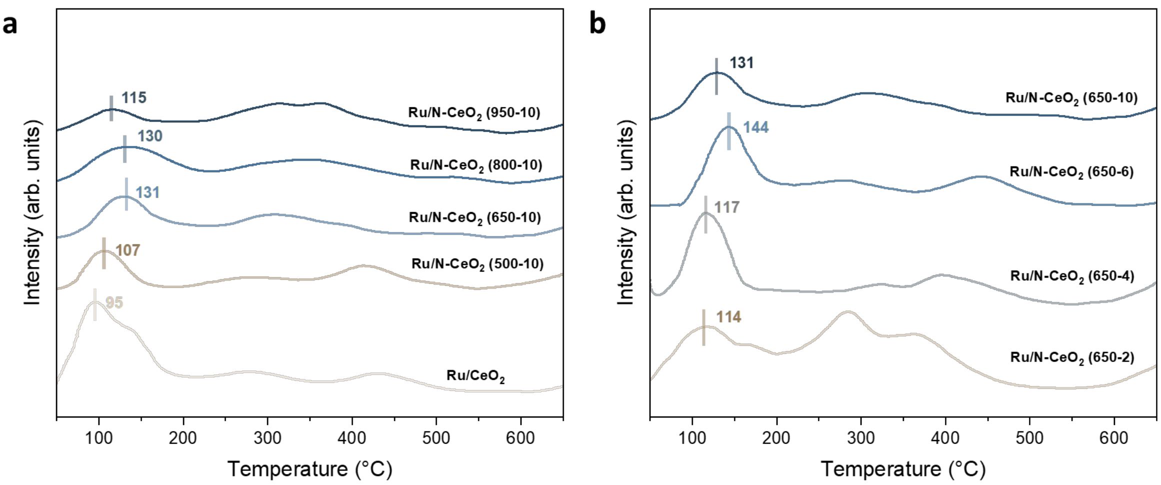

Fig. S11 H_2-TPR of Ru impregnated nitrogen doped cerium oxide. a. calcined at different temperature for 10 h b. calcined at 650 °C under different time scale.

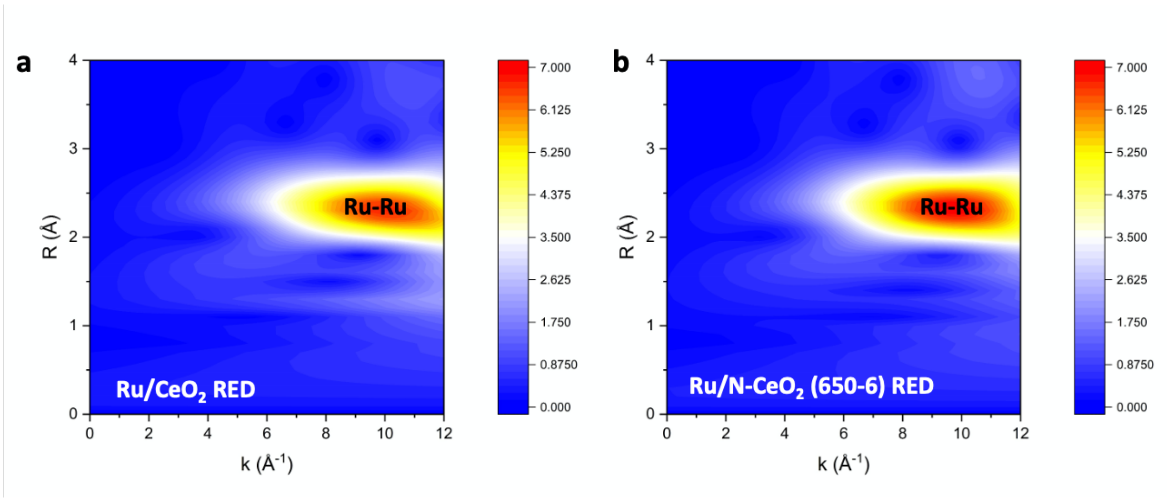

Fig. S12 Wavelet transformation plots for the k^3-weighted EXAFS signal of a. Ru/CeO_2 RED, maximum intensity at (9.82 $Å^{-1}$, 2.33 Å) and b. Ru/N-CeO_2 (650-6) RED, maximum intensity at (9.61 $Å^{-1}$, 2.33 Å).

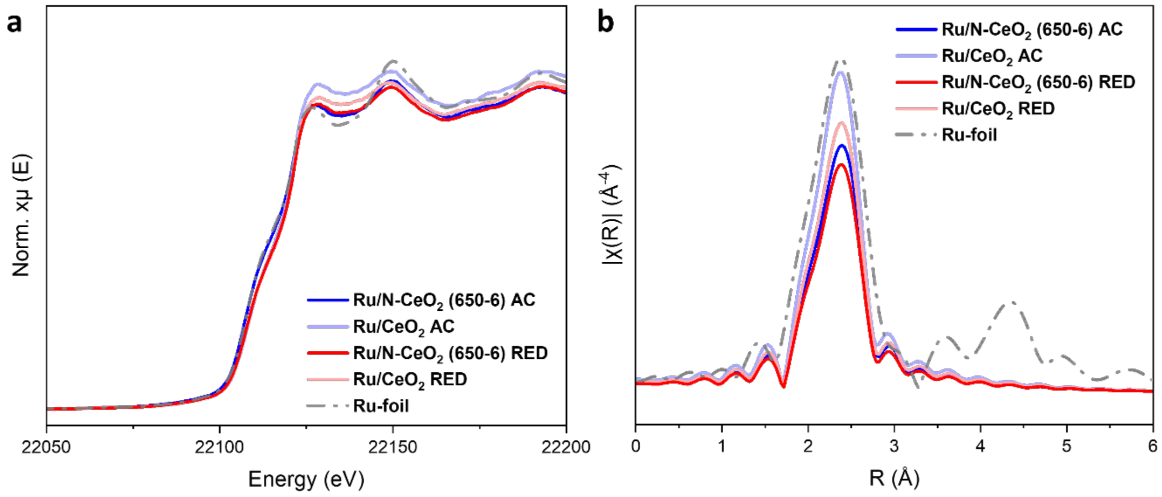

Fig. S13 X-ray adsorption spectroscopy of the analogue catalyst after reduction (RED) and after catalysis (AC) a. XANES and b. R-space from EXAFS.

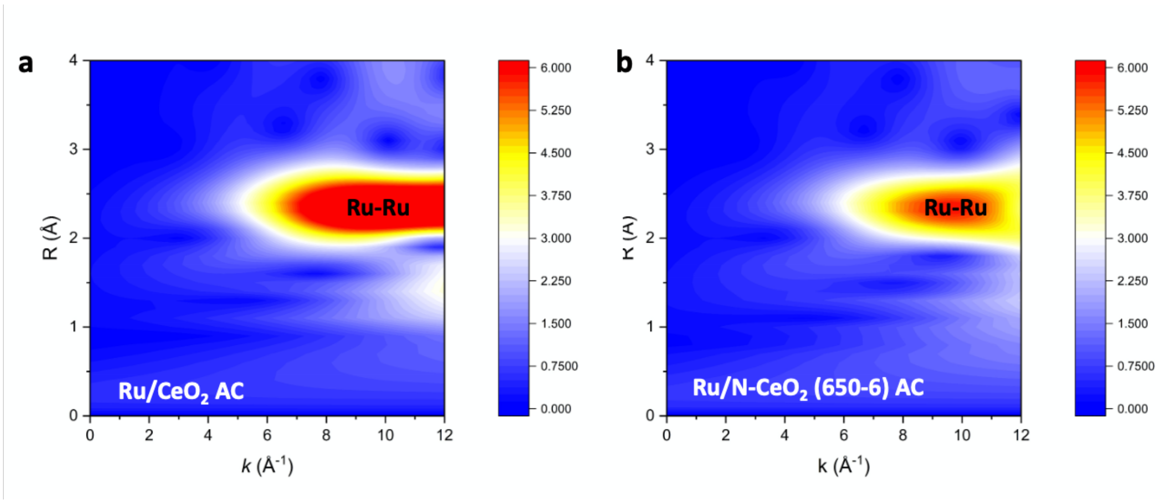

Fig. S14 Wavelet transformation plots for the k^3-weighted EXAFS signal of a. Ru/CeO_2 AC, maximum intensity at (10.84 $Å^{-1}$, 2.34 Å) and b. Ru/N-CeO_2 (650-6) AC, maximum intensity at (10.04 $Å^{-1}$, 2.34 Å).

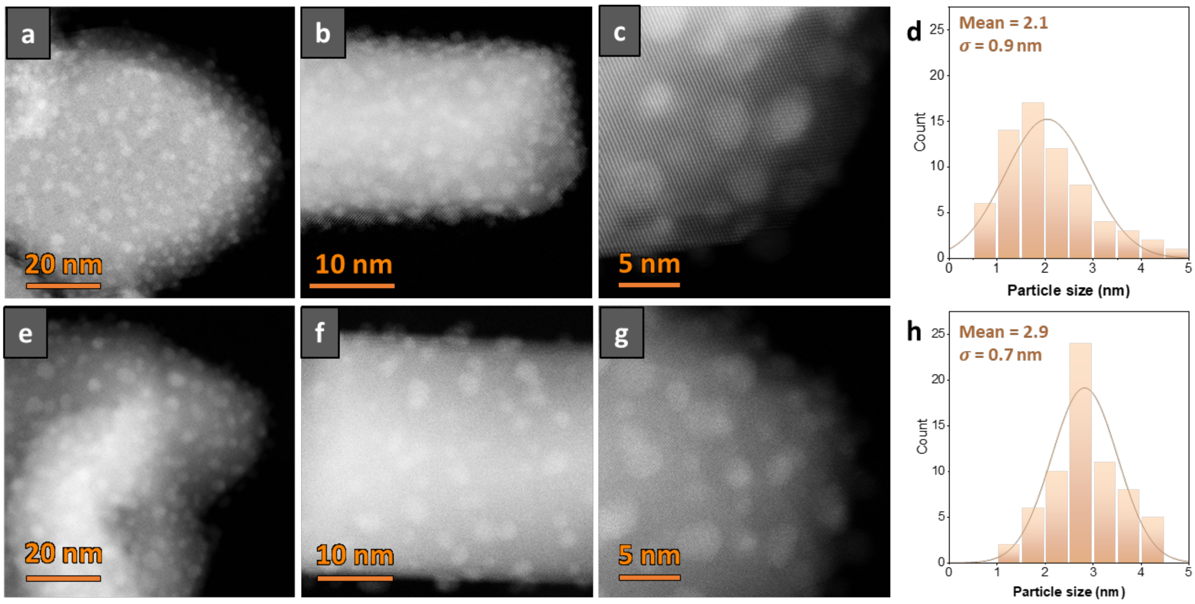

Fig. S15 TEM image and size distribution of Ru NPs on N-CeO_2 (650-6) support (a-d) and plain CeO_2 support (e-h).

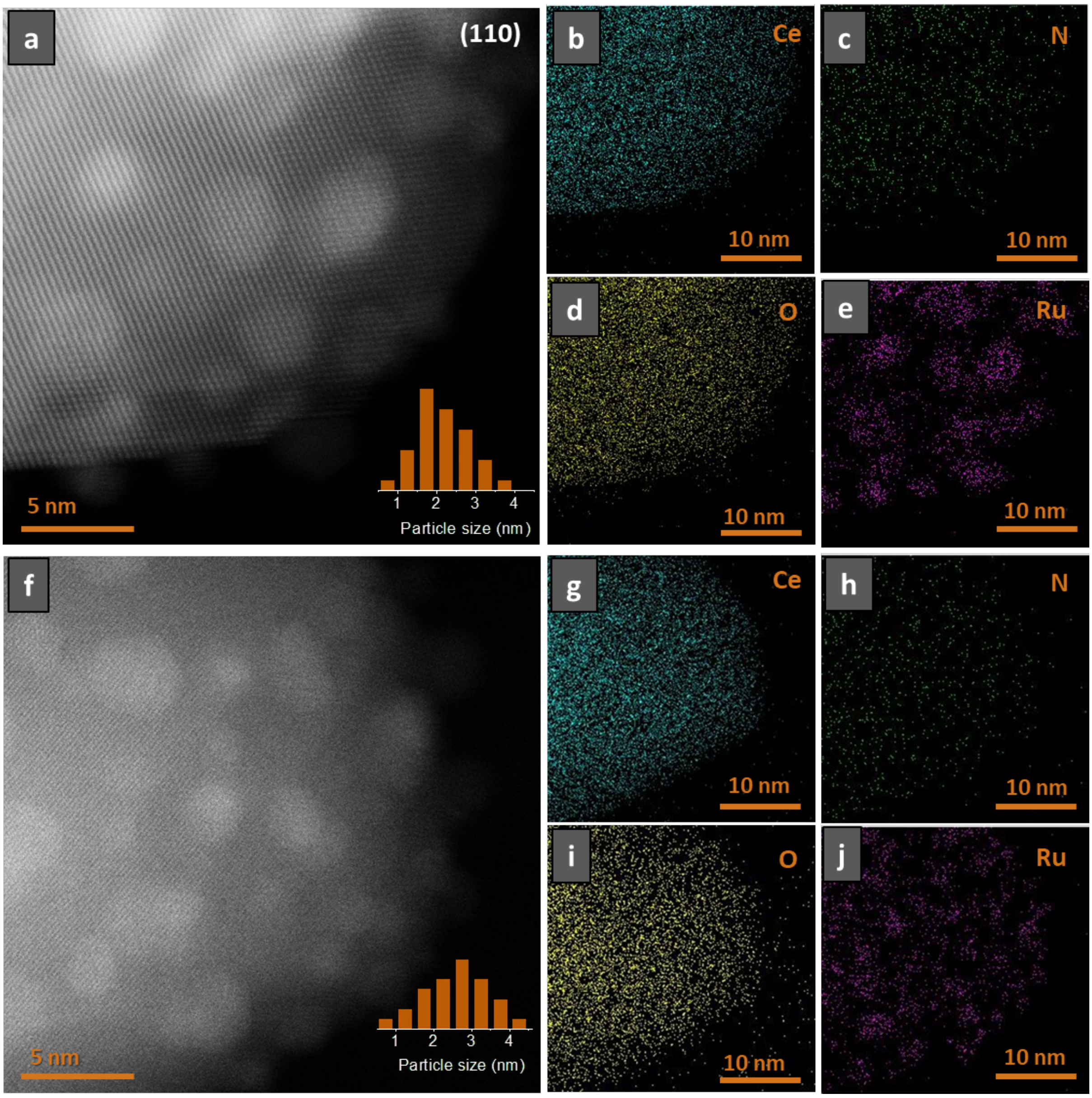

Fig. S16 Energy-dispersive X-ray spectroscopy (EDS) mapping on the Ce, N, O and Ru elements of b-e. Ru/N-CeO_2 (650-6) and g-j. Ru/CeO_2.

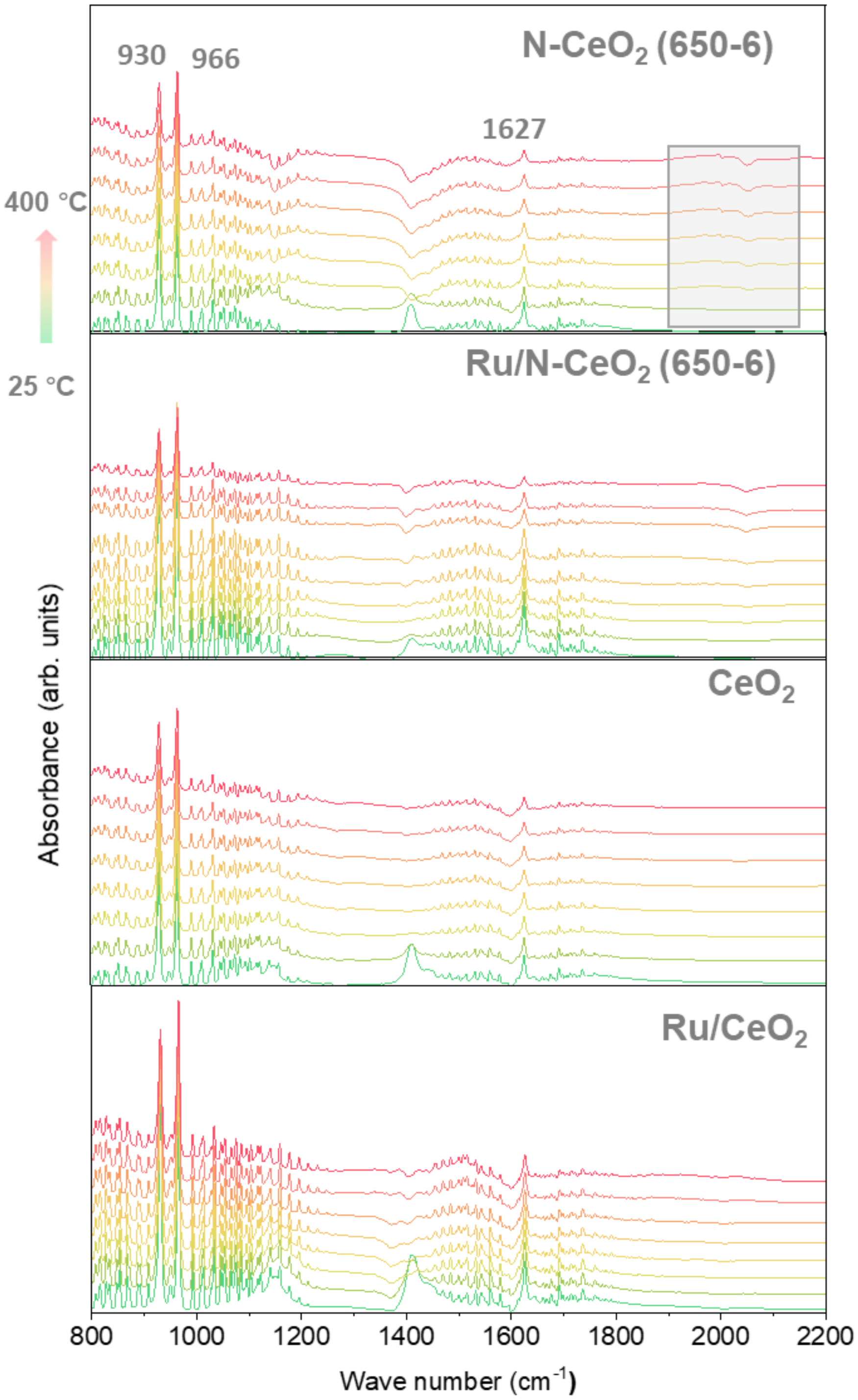

Fig. S17 *Operando* NH_3 FTIR for the analogue materials. The experiment was conducted at a range between 25 to 400 °C under continuous NH_3 flow. An enlarged figure of the shaded box illustrated in the above spectrum is presented in the main text as Fig. 3a.

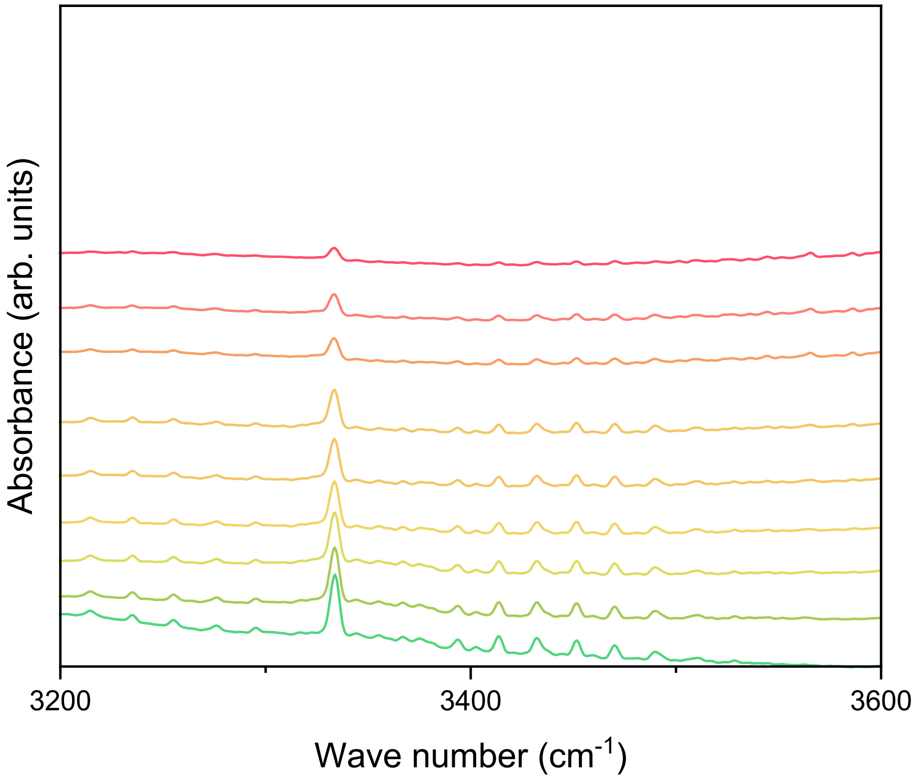

Fig. S18 *Operando* NH_3 FTIR of Ru/N-CeO_2 (650-6), focusing on the band at 3334 cm^{-1}. This feature corresponds to the N-H stretching mode of NH_3, arising from both gaseous NH_3 and NH_3 adsorbed on the catalyst surface. As the temperature increases, the intensity of this band decreases. Because the spectra were collected under a continuous flow of 10% NH_3/He, where the gas-phase NH_3 concentration is expected to remain constant, a reduction in this N-H signal indicates a genuine decrease in the total NH_3 population within the measurement cell. This behaviour is therefore consistent with NH_3 decomposition occurring at higher temperatures.

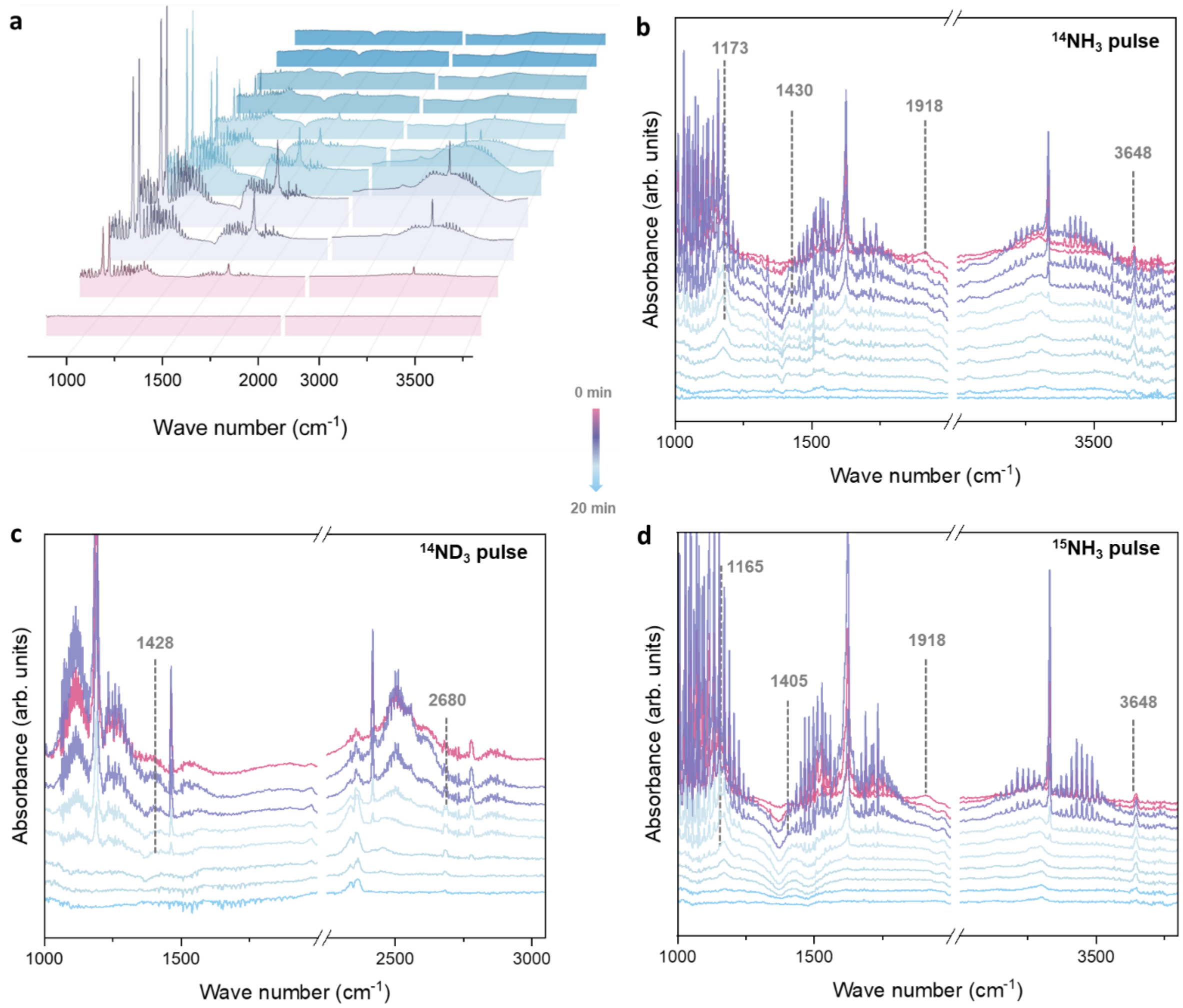

Fig. S19 Isotopic pulse experiment over *operando* FTIR on Ru/N-CeO_2 (650-6) at 400 °C and 10 ml min^{-1} Ar flush. Each isotopic pulse consists of 0.6 bar of reactant gas. a. 3D waterfall plot of time-resolved spectrum under $^{14}NH_3$ pulse b. Spectrum after a $^{14}NH_3$ pulse c. Spectrum after $^{14}ND_3$ pulse d. Spectrum after $^{15}NH_3$ pulse.

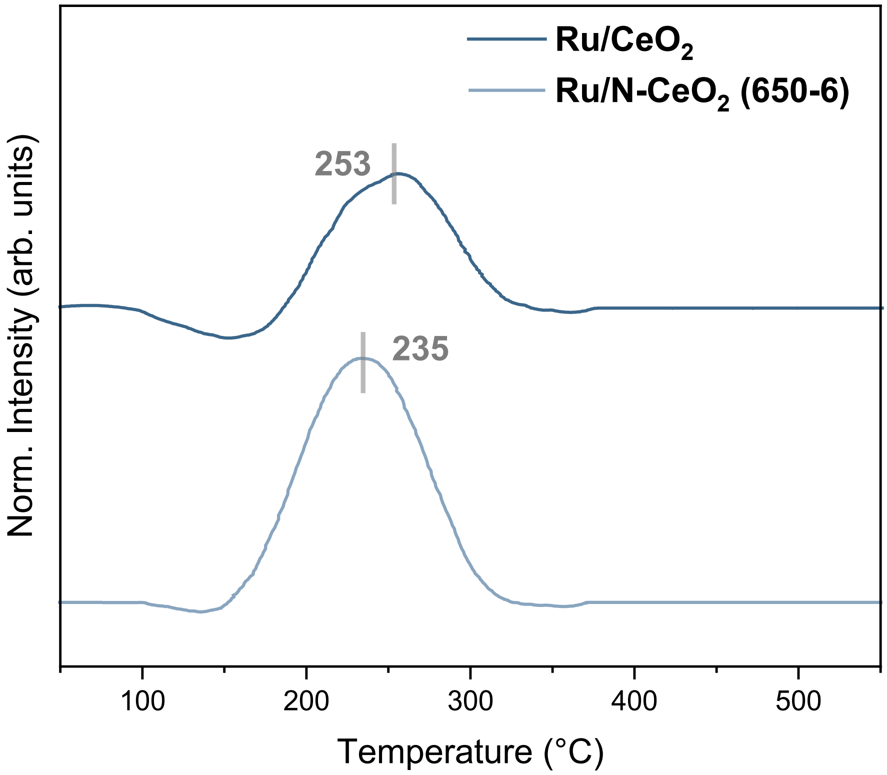

Fig. S20 NH_3-TPD experiment of Ru/CeO_2 and Ru/N-CeO_2 (650-6) catalysts. Only 1 peak centred in the range from 235-253 °C noted. Upon N doping, the catalyst exhibits a larger NH_3 adsorption capacity. This is in correspondence with the increased amount of oxygen vacancies observed from XPS. Furthermore, N dopants also serve as binding sites to uptake more NH_3 molecules. A shift in the desorption temperature for the N doped catalyst suggest that overall N binding energy of the catalyst is slightly lower compared to the undoped counterpart.

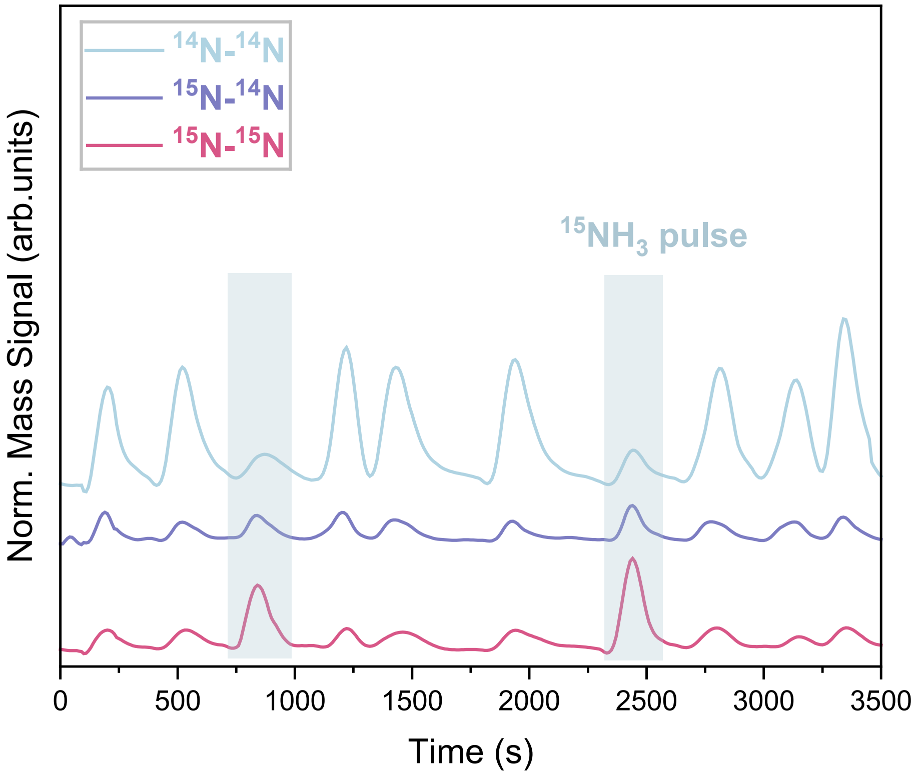

Fig. S21 $^{15}NH_3$/$^{14}NH_3$ pulse experiment of Ru/CeO_2 at 450 °C, 10 ml min^{-1} flow of Ar; each pulse contains 0.6 bar of either $^{15}NH_3$ or $^{14}NH_3$.

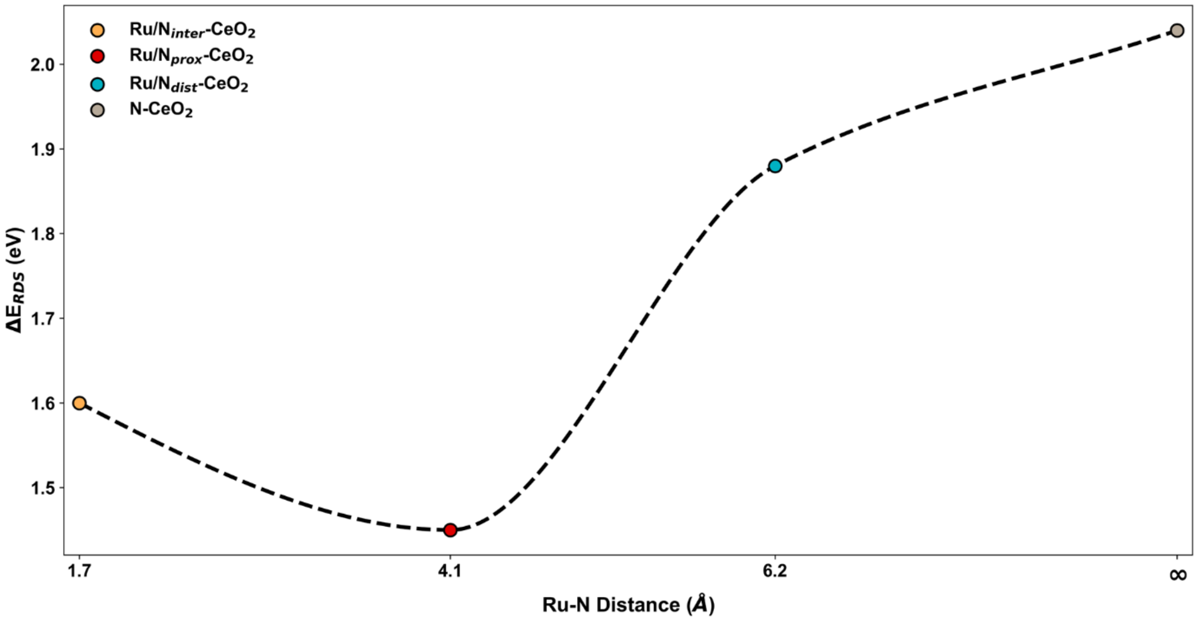

Fig. S22 Ru–N$_{doped}$ distance–dependent modulation of the rate-determining step barrier of MvK type NH_3 decomposition on Ru/N-CeO_2.

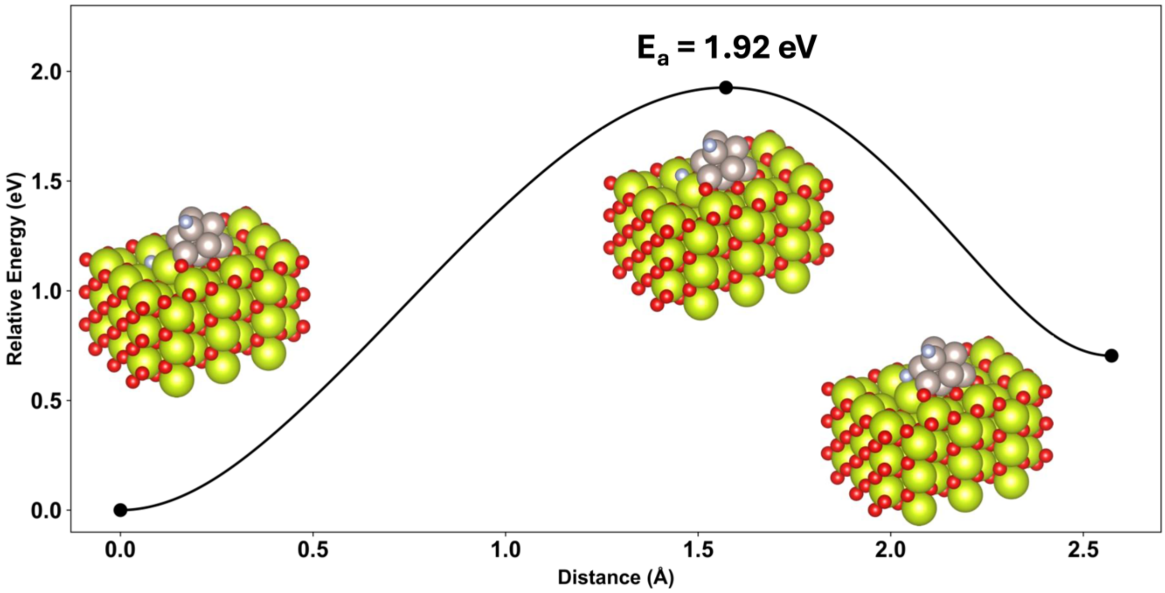

Fig. S23 Calculated CI-NEB result providing evidence for the independence of the pathways of NH_3 decomposition at CeO_2-proximal N versus at Ru cluster sites, due to a high migration barrier. The energy barrier for proximal N (doped at the CeO_2 surface) to migrate onto a site adjacent Ru cluster is critically high, calculated as 1.92 eV. This energy penalty effectively prohibits the dynamic coupling between the two distinct active sites—Ru cluster and proximal N—forcing them to operate independently during catalysis.

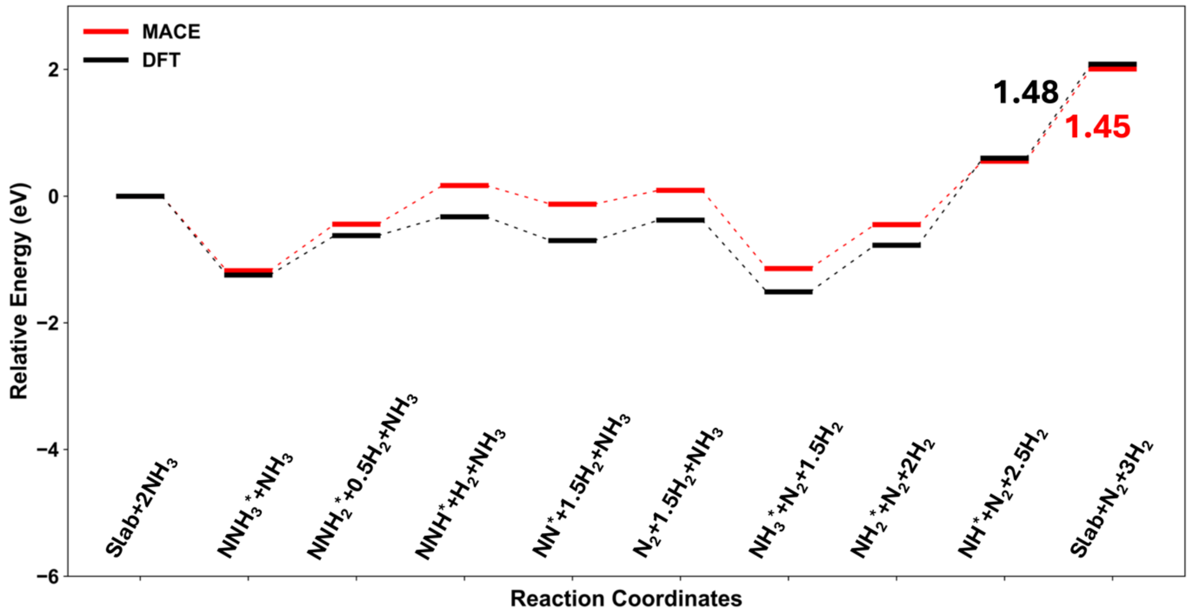

Fig. S24 Comparison between MACE- and DFT-calculated potential energy diagrams for the MvK type reaction on the proximal N site.

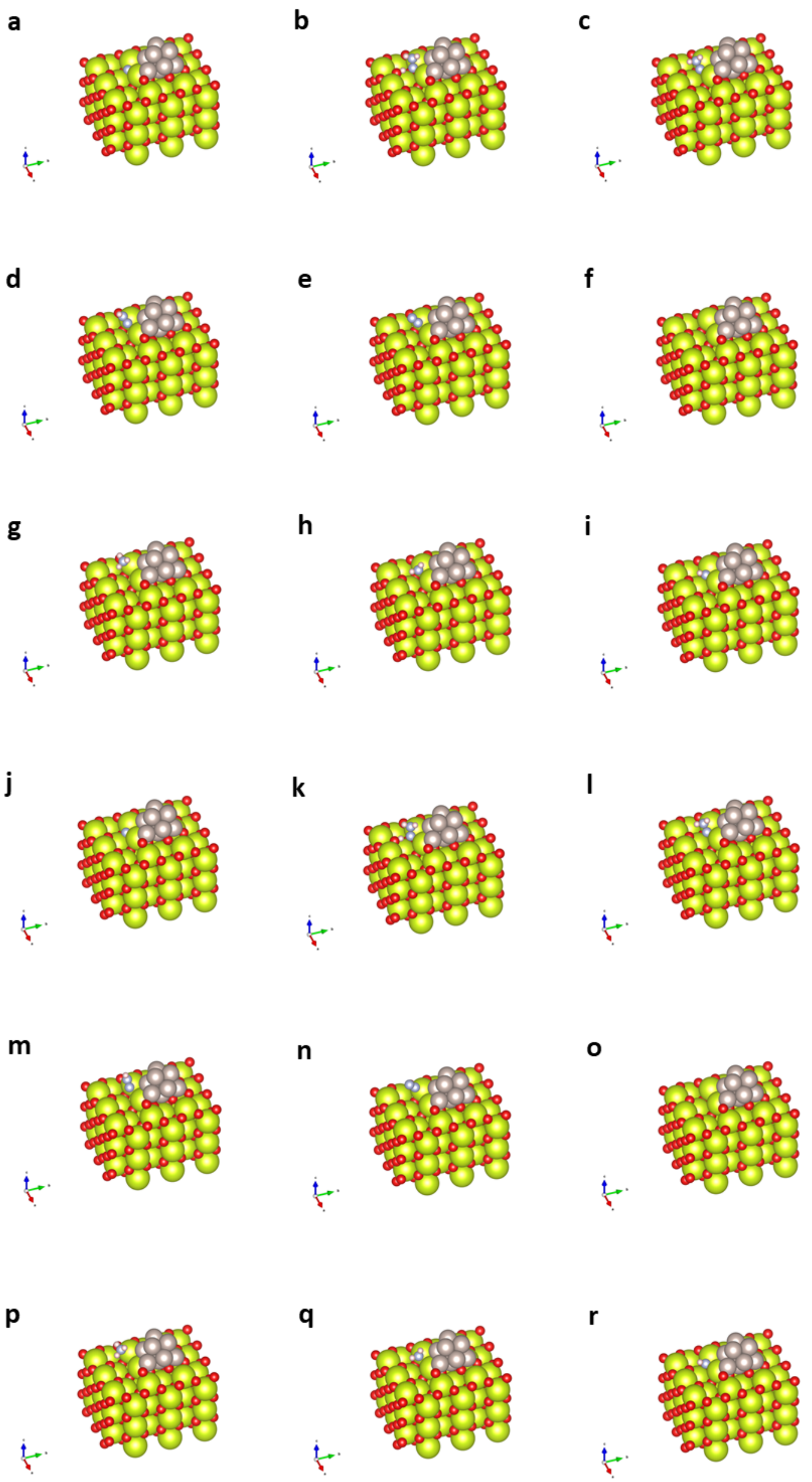

Fig. S25 Optimised structures along the MvK pathway, obtained by MACE (a-i) and DFT (j-r).

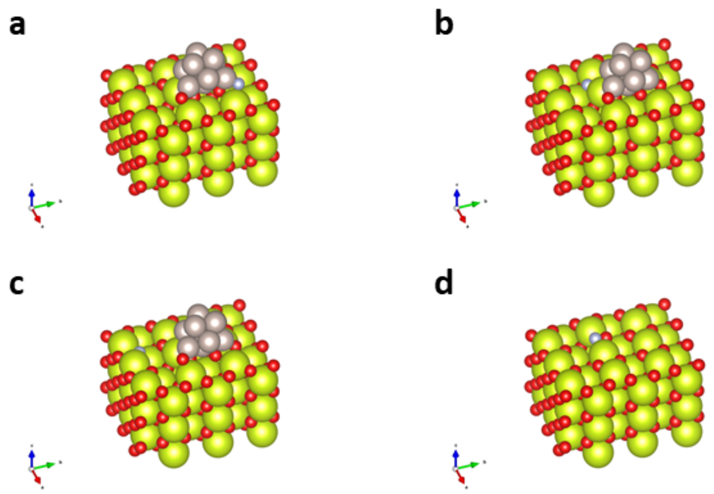

Fig. S26 MACE-optimised slab models. a. Ru/N_{int}-CeO_2. b. Ru/N_{pro}-CeO_2. c. Ru/N_{distal}-CeO_2. d. N-CeO_2.

Table S1 structural and elemental analysis of the analogue cerium-based supports

	CHNS analysis	XPS analysis		BET analysis
Sample	N content / %	N content / %	$Ce^{3+}/(Ce^{3+}+Ce^{4+})$ / %	Surface area / $m^2\ g^{-1}$
CeO_2	n/a	n/a	15.55	12.76
N-CeO_2 (500-10)	0.84	n/a	15.48	10.50
N-CeO_2 (650-10)	2.76	2.57	16.92	10.47
N-CeO_2 (800-10)	2.28	2.41	19.86	7.36
N-CeO_2 (950-10)	1.32	1.68	26.16	4.89
N-CeO_2 (650-2)	1.32	n/a	13.95	10.52
N-CeO_2 (650-4)	1.92	2.03	18.49	9.83
N-CeO_2 (650-6)	3.12	3.76	18.29	11.36

Table S2 Catalytic activity comparison with non-noble-metal based catalysts for ammonia decomposition.

Catalyst	Metal content /wt. %	Reaction Temp. /°C	WHSV /ml g_{cat}^{-1} h^{-1}	NH_3 Conv. /%	Reference
K–$CoNi_{alloy}$–MgO–CeO_2–SrO	60.0	450	12,000	87.5	[2]
CeO_2/Ni	60.0	500	30,000	72.4	[3]
Co@$BaAl_2O_4$	40.0	450	30,000	66.6	[4]
$LaCoO_x$/Co@NC/SBA-15	14.1	450	30,000	59.0	[5]
Mo_2N/SBA-15/rGO	25.3	450	30,000	31.0	[6]
Fe-Co/MgO	74.0	500	7,200	ca. 47.0	[7]
Co-Ba/Y_2O_3	29.7	450	30,000	ca. 50.0	[8]
Ru/CeO_2	0.15[a]	450	30,000	21.3	This work
Ru/CeO_2	0.31[a]	450	30,000	28.6	This work
Ru/CeO_2	0.91[a]	450	30,000	34.6	This work
Ru/CeO_2	3.23[a]	450	30,000	58.0	This work
Ru/N-CeO_2 (650-2)	0.15[a]	450	30,000	26.4	This work
Ru/N-CeO_2 (650-2)	0.31[a]	450	30,000	30.9	This work
Ru/N-CeO_2 (650-2)	0.91[a]	450	30,000	44.3	This work
Ru/N-CeO_2 (650-2)	3.23[a]	450	30,000	61.2	This work
Ru/N-CeO_2 (650-4)	0.15[a]	450	30,000	29.0	This work
Ru/N-CeO_2 (650-4)	0.31[a]	450	30,000	33.3	This work
Ru/N-CeO_2 (650-4)	0.91[a]	450	30,000	50.7	This work
Ru/N-CeO_2 (650-4)	3.23[a]	450	30,000	76.2	This work
Ru/N-CeO_2 (650-6)	0.15[a]	450	30,000	29.7	This work
Ru/N-CeO_2 (650-6)	0.31[a]	450	30,000	38.1	This work
Ru/N-CeO_2 (650-6)	0.91[a]	450	30,000	54.9	This work
Ru/N-CeO_2 (650-6)	3.23[a]	450	30,000	82.7	This work

Note: [a] Ru content of this work obtained using ICP-MS.

Table S3 EXAFS fitting parameters, related to Fig. 2b-e and S12-14

Composition	Shell	N	R (Å)	σ^2	R factor
Ru CeO_2 AS	Ru-O	5.931 ± 0.81	2.043 ± 0.017	0.0087	1.92%
Ru N-CeO_2 AS	Ru-O/N	6.729 ± 0.80	2.014 ± 0.015	0.0095	1.41%
Ru CeO_2 RED	Ru-Ru	9.466 ± 1.36	2.670 ± 0.006	0.0042	1.28%
Ru N-CeO_2 RED	Ru-Ru	8.460 ± 1.40	2.670 ± 0.005	0.0043	1.67%
Ru CeO_2 AC	Ru-Ru	11.138 ± 1.59	2.670 ± 0.005	0.0043	1.26%
Ru N-CeO_2 AC	Ru-Ru	8.516 ± 1.18	2.670 ± 0.005	0.0047	1.22%

N, coordination number; R (Å), bond length; σ^2, Debye-Waller factor

Table S4 Formation energy of the doped N site

Composition	N_{doped} site Formation Energy (eV)
Ru/N_{inter}-CeO_2	0.874
Ru/N_{prox}-CeO_2	1.916
CeO_2	3.352

Note: The formation energies were calculated as: $E_F = E_T(N_{doped}) + 1.5E(H_2) – E_T(O_v) – E(NH_3)$, where E_F denotes the formation energy, E_T is the total energy of the corresponding system.

Supplementary discussion 1: Fundamental structural characterisations.

The structure of the bulk support is analysed by powder X-ray diffraction (XRD). As illustrated in Fig. S1, the synthesized CeO_2 exhibits nine peaks at 2θ of 28.20°,32.74°, 47.16°, 56.05°, 58.79°, 69.16°, 76.44°, 78.82° and 88.20° which are well assigned to the (111), (200), (220), (311), (222), (400), (331), (420) and (422) planes of CeO_2, respectively.[9] This XRD pattern matches with the face-centred cubic structure of CeO_2 (PDF# 34-0394), indicating a fluorite structure and a crystalline cubic $Fm\bar{m}3m$ space group. The N-CeO_2 class of catalysts show nearly identical diffraction pattern reflecting no change in the fluorite structure after the NH_3 treatment. Higher crystallinity was observed for N-CeO_2 synthesized at higher temperature, indicated by sharper diffraction peaks. This is attributed to the elimination of grain boundary defects during calcination at high temperatures, resulting in increasing the crystallinity of the N-CeO_2 nanoparticles. Furthermore, the crystal size of the samples increases with higher calcination temperature. As revealed by scanning electron microscopy (SEM) (Fig. S2), the CeO_2 support shows featureless and amorphous structures with few crystallites of size ca. 100 nm. After annealing in NH_3, the structure becomes more ordered and aggregation of crystallites was observed. At higher temperature, even under reduction gas flow, crystal growth is induced via Ostwald ripening, smaller particles tend to merge to reduce surface energy and form more thermodynamically stable crystallites.[10] This is also evidenced by the decrease in surface area determined through BET analysis (Table S1).

Supplementary discussion 2: Optical presentation of the materials.

Alteration in the NH_3 treatment condition during the N doping step results in different sample colours observed for the finally obtained powder material. The pale yellow CeO_2 is turned light grey after annealing in NH_3 at 500 °C (N-CeO_2 (500-10)). At higher synthesis temperatures, it further turns pale grey (N-CeO_2 (650-10)), light blue (N-CeO_2 (800-10)) and finally olive green (N-CeO_2 (950-10)).

Supplementary discussion 3: Further explanation of the catalytic results.

In general, a higher catalytic activity is observed with higher N dopant level. However, it is noteworthy that the conversion of Ru/N-CeO_2 (500-10) shows comparative activity to Ru/N-CeO_2 (800-10) given that the nitrogen content of the latter is much higher. This is due to the increase in crystal size observed from both XRD and SEM data (Fig. S1-2). Larger crystal size is always accompanied by lower surface area to increase thermodynamic stability, thereby worsening the dispersion and leading to growth of larger Ru NPs, which is another key factor reducing the catalytic activity.[11,12] The promotion effect from extra N incorporation thus cancels out and therefore leads to the volcano-shaped trend presented.

Supplementary discussion 4: Effect of Ru loading versus N content towards the catalytic activity

In general, the catalytic conversion of NH_3 increases with either higher N-doping levels or higher Ru loadings. For Ru loading, increasing the number of Ru nanoparticles leads to a larger population of Ru B_5 active sites, thereby enhancing the overall reaction rate. The effect of N doping is slightly different. As discussed in the main text, N dopants not only promote the intrinsic activity of Ru but can also serve as independent active sites that facilitate the associative reaction mechanism. Thus, increasing the N-dopant concentration also enhances catalytic activity.

As shown in Fig. S7, the NH_3 conversion achieved by Ru/N-CeO_2 (650-6) (Ru content: 0.3 wt.%; N content: 3.1 at.%) is ca. 38%, which is comparable to that of Ru/CeO_2 (Ru content: 0.9 wt.%; N content: 0 at.%), ca. 35%. This demonstrates that introducing N dopants can reduce the required Ru loading by nearly threefold while maintaining similar catalytic performance.

It is also notable that the promotional effect of N dopants is more pronounced at lower Ru loadings. At low Ru loadings, a greater fraction of the support surface remains exposed, allowing N-doped sites to contribute more significantly to the associative pathway. In contrast, at higher Ru loadings, increased coverage of the support surface by Ru nanoparticles decreases the number of accessible N active sites. As a result, the catalytic activity becomes dominated by the Ru B_5 sites, where the reaction primarily follows the dissociative mechanism, and the promotional influence of N dopants is diminished.

Supplementary discussion 5: *Operando* FTIR analysis.

Four characteristic NH_3 vibrational features are observed in the FTIR spectra: two symmetric N-H bending modes at 930.5 and 966.6 cm^{-1}, an asymmetric N-H bending mode at 1627 cm^{-1}, and a symmetric N-H stretching mode at 3334 cm^{-1} (Figs. S17–S18).[13] The FTIR measurements were conducted under *operando* conditions, with the catalyst continuously purged by 10 mL min^{-1} of 10% NH_3/He. Under these conditions, the 3334 cm^{-1} band reflects contributions from both gaseous NH_3 and NH_3 adsorbed on the catalyst surface, with the gaseous-phase component dominating the overall intensity. The intensity of the 3334 cm^{-1} band decreases for Ru-impregnated catalysts as temperature increases (Fig. S18). This attenuation cannot be attributed to NH_3 desorption, as desorption alone would not alter the total population of N-H species in the measurement cell. Instead, the decline in intensity signifies the onset of NH_3 decomposition, in which NH_3 is converted to N_2 and H_2, reducing the number of N-H species contributing to the signal. This temperature-dependent behaviour aligns with reports from the literature on thermal catalytic NH_3 decomposition.[14] Additional evidence supporting NH_3 decomposition is provided by the appearance of a broad O-H band during the $^{14}NH_3$-pulsed FTIR experiments at elevated temperatures (Fig. 3b). The formation of hydrogen during NH_3 decomposition facilitates the generation of surface hydroxyl species through reaction with lattice oxygen, producing O-H vibrational features that diminish upon purging, consistent with hydroxyl formation. At lower temperatures, a sharp band at 1400-1425 cm^{-1} is observed, corresponding to NH_3 adsorption on CeO_2 surface sites.[15,16]

It should be noted that the *operando* FTIR cell used in this study has a maximum operating temperature of 400 ºC, which defines the upper limit of the measurements. The high catalyst mass required (~200 mg) and relatively low flow rate (10 mL min^{-1} of 10% NH_3/He) correspond to a weight hourly space velocity (WHSV) of ~3000 mL g^{-1} h^{-1}, much lower than that used in catalytic performance tests (15,000 mL g^{-1} h^{-1}). Under such low WHSV conditions, increasing the temperature above 400 ºC would likely result in near-complete NH_3 conversion, making the detection of intermediates and mechanistic features exceedingly difficult. For this reason, *operando* FTIR measurements at temperatures above 400 °C are not suitable for mechanistic analysis in this system, as the NH_3 stretching band at 3334 cm^{-1} would no longer be observable. The trends observed in Figs. S17-S18 reliably demonstrate the enhanced decomposition of NH_3 at elevated temperatures and support the mechanistic conclusions discussed in the main text.

Supplementary discussion 6: Associative route analysis on Ru/N-CeO_2 (650-6) and N-CeO_2 (650-6).

It is noted that the peak intensity of the *NNH_x bonds is much smaller for the Ru-impregnated sample (Fig. S17). This is attributed to the following: highly dispersed Ru NPs covers the surface of the support, leading to reduced interaction between NH_3 and the lattice N; incorporation of Ru NPs would significantly accelerate the reaction, resulting in lower retention time on the support, observed via FTIR; Ru NPs would also adsorb NH_3 and perform NH_3 cracking via a dissociative mechanism. Under this reaction route, -N_2H is not an intermediate as the N triple bond is directly formed between two bare N atoms. Thus, this competition between the two routes would also result in a less significant peak change.

Supplementary References

1. Yan, X. *et al.* Cooperatively tailored surface frustrated Lewis pairs and N-doping on CeO_2 for photocatalytic CO_2 reduction to high-value hydrocarbon products. *Appl. Catal. B Environ.* **343**, 123484 (2024).
2. Tabassum, H. *et al.* Hydrogen generation via ammonia decomposition on highly efficient and stable Ru-free catalysts: approaching complete conversion at 450 °C. *Energy Environ. Sci* **15**, 4190–4200 (2022).
3. Liu, H., Zhang, R., Liu, S. & Liu, G. CeO_2/Ni Inverse Catalyst as a Highly Active and Stable Ru-free Catalyst for Ammonia Decomposition. *ACS Catal.* **14**, 9927–9939 (2024).
4. Xiong, P. *et al.* Synthesis of core@shell catalysts guided by Tammann temperature. *Nat. Commun.* **15**, 1–9 (2024).
5. Han, X. *et al.* Dual confinement of $LaCoO_x$ modified Co nanoparticles for superior and stable ammonia decomposition. *Appl. Catal. B Environ.* **328**, 122534 (2023).
6. Huo, L. *et al.* Spatial confinement and electron transfer moderating MoN bond strength for superior ammonia decomposition catalysis. *Appl. Catal. B Environ.* **294**, 120254 (2021).
7. Chen, S. *et al.* Highly loaded bimetallic iron-cobalt catalysts for hydrogen release from ammonia. *Nat. Commun.* **15**, 871- (2024).
8. Xu, K. *et al.* Single-Atom Barium Promoter Enormously Enhanced Non-Noble Metal Catalyst for Ammonia Decomposition. *Angew. Chem. Int. Ed.* **64**, e202416195, (2025).
9. Li, Y. *et al.* Roles of Oxygen Vacancies in CeO_2 Nanostructures for Catalytic Aerobic Cyclohexane Oxidation. *ACS Appl. Nano Mater.* **6**, 14214–14227 (2023).
10. Kim, M. G. *et al.* Effects of Calcination Temperature on the Phase Composition, Photocatalytic Degradation, and Virucidal Activities of TiO_2 Nanoparticles. *ACS Omega* **6**, 10668–10678 (2021).
11. Bueno-Ferrer, C., Parres-Esclapez, S., Lozano-Castelló, D. & Bueno-López, A. Relationship between surface area and crystal size of pure and doped cerium oxides. *J. Rare Earths* **28**, 647–653 (2010).
12. Zhou, Y. *et al.* Unraveling the size-dependent effect of Ru-based catalysts on Ammonia synthesis at mild conditions. *J. Catal.* **404**, 501–511 (2021).
13. Kim, J. S., Nam, H., Kim, H. J., Lee, J. H. & Park, B. H. Real-Time Measurement of Ammonia (NH_3) in Artillery Smoke Using a Passive FT-IR Remote Sensor. *ACS Omega* **4**, 16768–16773 (2019).
14. Boisen, A., Dahl, S., Nørskov, J. K. & Christensen, C. H. Why the optimal ammonia synthesis catalyst is not the optimal ammonia decomposition catalyst. *J. Catal.* **230**, 309–312 (2005).
15. Duan, C. Peng, Guo, R. Tang, Wu, G. Lin & Pan, W. Guo. Selective catalytic reduction of NO_x by NH_3 over $CeVO_4$-CeO_2 nanocomposite. *Environ. Sci. Pollut. Res.* **27**, 22818–22828 (2020).
16. Qi, G. & Yang, R. T. Characterization and FTIR studies of MnO_x-CeO_2 catalyst for low-temperature selective catalytic reduction of NO with NH_3. *J. Phys. Chem. B* **108**, 15738–15747 (2004).
